# Microencapsulation by Spray Drying and Antioxidant Activity of Phenolic Compounds from Tucuma Coproduct (*Astrocaryum vulgare* Mart.) Almonds

**DOI:** 10.3390/polym14142905

**Published:** 2022-07-18

**Authors:** Lindalva Maria de Meneses Costa Ferreira, Rayanne Rocha Pereira, Fernanda Brito de Carvalho-Guimarães, Myrth Soares do Nascimento Remígio, Wagner Luiz Ramos Barbosa, Roseane Maria Ribeiro-Costa, José Otávio Carréra Silva-Júnior

**Affiliations:** 1 Laboratory of Pharmaceutical and Cosmetic R&D, Federal University of Pará, Belém 66075-110, Brazil; lindalva.costa.ferreira@ics.ufpa.br (L.M.d.M.C.F.); rayannerocha@yahoo.com (R.R.P.); 2 Laboratory of Innovation and Development of Pharmaceutical Technology, Federal University of Amazonas, Manaus 69067-005, Brazil; fernandabc_@hotmail.com; 3 Laboratory of Phytochemistry, Federal University of Pará, Belém 66075-110, Brazil; myrth.fa@hotmail.com (M.S.d.N.R.); barbosa@ufpa.br (W.L.R.B.); 4 Laboratory of Nanotechnology Pharmaceutical, Federal University of Pará, Belém 66075-110, Brazil; rmrc@ufpa.br

**Keywords:** phenolic compounds, agroindustrial coproduct, tucuma, microencapsulation, spray drying

## Abstract

The industrial processing of fruits in the Amazon region, such as tucuma, generates a large amount of coproducts with great nutritional potential. In this work, phenolic compounds from tucuma coproduct almonds were extracted and microencapsulated by spray drying using maltodextrin as the encapsulating agent and verified its antioxidant activity. Phenolic compounds were determined by UV spectroscopy and identified by Ultraefficiency Liquid Chromatography. Antioxidant activity was measured by ABTS and DPPH assay. Thermogravimetric techniques, infrared spectroscopy, scanning electron microscopy, moisture content and water activity were applied to characterize the microparticle. The crude extract and microparticle had total polyphenols of 135.1 mg/g ± 0.078 and 130.5 mg/g ± 0.024, respectively. Caffeic and gallic acids were identified. The crude extract and the microparticle showed good antioxidant activity by ABTS and DPPH assay, justified by the presence of the phenolic compounds found. The microparticle showed spherical and heterogeneous structures and good encapsulation efficiency from the spray drying process using maltodextrin. The results show that the extract of the tucuma almond coproduct can be used as a phenolic compound-rich source and microencapsulated with possible application for functional food production.

## 1. Introduction

The Amazon region harbors a great biodiversity of plant species that produce fruits and oilseeds with economic, technological and nutritional potential, highlighting their use and applications in the food and cosmetics industry. Tucuma (*Astrocaryum vulgare* Mart.) is a palm belonging to the Arecaceae family, found in northern Brazil [1]. It has an oleaginous fruit whose mesocarp is fibrous and yellow–orange, nutritious and rich in lipids and compounds such as provitamin A [2,3,4]. Tucuma has many uses, popularly, the pulp of the fruit is consumed in natura. The food industry uses pulp in the preparation of creams and ice creams. The cosmetic industry uses the pulp of tucuma for extraction of the oil, used mainly for skin benefits (photoprotection and hydration) [5,6]. These processes generate a large amount of coproducts that are discarded in the environment without any prior treatment. After the pulp and oil is obtained, the tucuma seed is discarded in tons each year [7]. Each month, 83 tons of fruit are sold, of which 21 tons are tucuma pulp (peeled manually) and about 60% are waste; 50 tons are disposed of in the trash [8].

The coproducts generated from the processing of fruits, such as seeds, grains and bagasse have great potential for reuse because they present a significant concentration of phenolic compounds (phenolic acids, flavonoids and tannins) and carotenoids [3,9,10]. Phenolic compounds are of interest due of their antimicrobial, anticarcinogenic, antitumorigenic and antioxidant properties [11]. The antioxidant activity found in the tucuma coproduct is related to the presence of phenolic acids (gallic acid and caffeic acid). The antioxidant activity of phenolic acids is mainly related to the reducing properties of their structure, which acts both in capturing reactive species and chelating transition metals, and thus participates in the initiation and propagation stages of the oxidative process [12]. The extraction of bioactive compounds from the fruit coproduct can increase the profitability of the process and valorize the raw material [3,9,13].

However, these compounds, when exposed to extreme values of pH, temperature, light, oxygen and enzymes, can suffer degradation reactions such as oxidation that influence the loss of their bioactivity [14,15]. Microencapsulation consists of incorporating the substances of interest (core or active material) into a coating system (wall material, carrier or encapsulating agent), generating microparticulate systems or spherical microparticles with sizes that can vary from 1 to 1000 µm [16,17]. In this perspective, microencapsulation by spray drying becomes a promising alternative for protecting the bioactive compounds using a polymeric matrix [3,18,19,20]. Spray drying is an encapsulation technique widely used in the food industry [21,22,23] and consists of the atomization of a liquid product into a stream generating a pulverized product of warm air [24,25]. Due to solvent removal, spray drying guarantees microbiological stability, avoids degradation processes, reduces storage and transport costs and improves the solubility of the final product [26,27,28].

Among the factors responsible for the success of the microencapsulation process is the choice of the encapsulating agent [29,30]. Encapsulating agents can be of natural, semi-synthetic or synthetic origin, including polymeric, hydrophilic, hydrophobic materials or a combination of both; their function is to provide protection during prolonged storage, preventing chemical and sensory changes from occurring in the encapsulated material [31]. The polysaccharide maltodextrin obtained by the acid hydrolysis of the starch is widely used by the food industry as an adjuvant in spray drying [32]. This has promising properties for microencapsulation, among them are good film forming and emulsifying properties, low hygroscopicity, low viscosity at high solid concentrations, mild taste and odor and low cost [33]. In action as an encapsulating agent in plant extracts, it increases the glass transition temperature (Tg) of unstable compounds or substances with high sugar content [32].

Amazonian fruit species such as tucuma arouse interest in study due to their economic potential due to their vast popular use. Ferreira, L. et al. [3] obtained the oily extract of the coproduct of tucuma almonds by green extraction using palm oil by the ultrasound method and then carried out microencapsulation by spray dryer and verified its antioxidant activity. However, the importance of studies using the agroindustrial coproduct of tucuma is seen due to its high content of phenolic compounds and antioxidant capacity which allocate it as a suitable destination in a rational and sustainable way. In this sense, knowing the chemical composition and antioxidant potential of the coproduct of tucuma almonds from the industry can minimize the environmental impacts caused by not using them and is an option as a way of adding value to the production chain. Thus, the objective of this study was to obtain the extract from the coproduct of the tucuma kernels and then microencapsulate it by spray drying using maltodextrin as the encapsulant agent. In the same way, this study evaluated their physical–chemical characteristics, quantified the content of phenolic compounds by spectrophotometry and identified the majority by Ultrahigh-Performance Liquid Chromatography (UHPLC) and their antioxidant capacity. It should be emphasized that this was the first time the content of phenolic compounds was extracted from the coproduct of tucuma almonds and that microencapsulation of these compounds from the coproduct by spray drying was performed. Therefore, this study is the first study to detect phenolic acids and significant antioxidant activity of the tucuma coproduct.

## 2. Materials and Methods

### 2.1. Chemicals, Reagents and Encapsulating Agents

Gallic acid, quercetin, epicatechin, hydrated rutin, 2-hydroxycinnamic caffeic acid, 2,2-diphenyl-1-picrylhydrazyl (DPPH), 2,2-azinobis (3-ethylbenzothiazoline-6) sulfonic acid) (ABTS) and 6-Hydroxy-2,5,7,8-tetramethylchroman-2-carboxylic acid (Trolox) were purchased from Sigma-Aldrich (St. Louis, MI, USA). Potassium persulphate, Folin-Ciocalteu, sodium carbonate (Na_2_CO_3_), vanillin, aluminum chloride and potassium bromide (KBr) were purchased from LabSynth (São Paulo, Brasil). The encapsulating agent maltodextrin (MD) with dextrose equivalent (DE 10) was purchased from Corn Products (São Paulo, Brazil).

### 2.2. Sample Preparation

Tucuma (*Astrocaryum vulgare* Mart.) seeds were submitted to a moderate heating process at 65 °C for 45 min, after which they were pressed to remove the oil to be industrially exploited. The resulting coproduct, designated as tucuma seed coproduct, was provided by the Amazon Oil Industry (Ananindeua, Brazil). After receiving it, the coproduct was packed and stored in a freezer (−18 °C). The material was dried for seven days in an oven with circulation and renewal of air (SL-102 SOLAB, Piracicaba, São Paulo, Brazil) at a temperature of 40 ± 2 °C. During the whole period that the material was submitted to the oven, it was followed until the constant weight was obtained in order to then determine the end of the drying period. After dehydration, the dried material was weighed and ground in a knife mill and the powder of the dry coproduct of the tucuma almonds was obtained and stored in a freezer (−18 °C) until the moment of use [3].

### 2.3. Extraction of Polyphenols from the Coproduct

To obtain the crude extract for extracting the polyphenols, the coproduct was placed in contact with an ethanol–water solution (70:30 *v*/*v*) in a stainless-steel percolator and subjected to extraction. The process was carried out by dispersing 1 Kg of the powder in 10 L of 70% hydroethanolic solution in the proportion of 1:10 (*p*/*v*) and left in maceration for 72 h. After this period, the percolation process was started with a dripping of 20 drops/min for a period of 5 days at room temperature. The extract was subjected to a rotary evaporator (Buchi R-210, Geneva, Switzerland) at controlled temperature (40 ± 2 °C) until complete solvent evaporation (ethanol) [34]. The extract-denominated crude extract (CE) was conditioned in an amber flask maintained under refrigeration (−18 °C) until the analysis.

### 2.4. Microparticle Preparation (MP)

Microparticles were obtained in two steps. Maltodextrin (MD) was the encapsulating agent. The first step was the homogenization of maltodextrin (5%) in the crude extract (5%) under magnetic stirring for 30 min at the final volume (400 mL). Then, it was dispersed to the UltraTurrax (IKA, T 125, São Paulo, Brazil) at 2500 rpm for 5 min for complete homogenization [35]. The next step was the atomization of the emulsion obtained using a spray dryer (LM-MSDi 1.0 Labmaq do Brasil–LTDA, São Paulo, Brazil), according to the parameters: temperature of 100 °C; flow rate of 7.5 mL/min and pressure of 6 bar. During the whole process, the emulsion was kept under agitation to ensure homogeneity. The microparticle obtained was called MP 5%.

### 2.5. Encapsulation Efficiency (EE)

The *EE* of the polyphenols was calculated as the content of polyphenols contained in the microparticle in relation to the content of polyphenols contained in the crude extract before drying [36].

(1) $$\%\;EE=\frac{\mathrm{content}\;\mathrm{of}\;\mathrm{polyphenols}\;\mathrm{in}\;\mathrm{the}\;\mathrm{microparticle}}{\mathrm{content}\;\mathrm{of}\;\mathrm{polyphenols}\;\mathrm{in}\;\mathrm{the}\;\mathrm{extract}\;\mathrm{before}\;\mathrm{drying}}\;\times 100$$

The polyphenols content was extracted from the MP 5% (0.4 g) with the addition of 2 mL of a methanol/acetic acid/water solution (50:8:42 *v*/*v*/*v*). The vortex dispersion was homogenized for 1 min and placed in an ultrasonic bath at a frequency of 42 KHz and a power of US:160 W (Cleaner Kondentech, São Paulo, Brazil) for 20 min. It was centrifuged for 15 min at 7500 rpm. The supernatant was filtered in membranes with a pore diameter of 0.45 µm (Millipore, Bedford, MA, USA) [37] for subsequent quantification of the total phenolic, total flavonoid and Tannin condensate content.

### 2.6. Quantification of Polyphenols by Spectrophotometry

#### 2.6.1. Total Phenolic Content (TPC)

The TPC quantification in the MP 5% was performed by the UV/Vis spectrophotometric method (Shimadzu UV 1800, Kyoto, Japan). Briefly, 100 μL of the solution obtained from the extraction of the polyphenols content from the microparticles was mixed with 500 μL Folin Ciocalteau reagent and 6 mL water solution incubated for 2 min. After, 2 mL of 10% saturated sodium carbonate was added and incubated in the dark for 2 h followed by measurement of absorbance at 760 nm. The TPC was calculated based on a standard curve of gallic acid (60–200 mg/mL) expressed as mg gallic acid equivalent (mg GAE/100) [38]. The analysis was also performed on the crude extract before drying in the same way that was performed on the microparticle.

#### 2.6.2. Total Flavonoid Content (TFC)

The TFC quantification in the MP 5% was performed by the UV/Vis spectrophotometric method (Shimadzu UV 1800, Kyoto, Japan). Briefly, 800 μL of the solution obtained from the extraction of polyphenols content from the microparticles was mixed with 400 μL 2.5% aluminum chloride solution and incubated in the dark for 30 min followed by measurement of absorbance at 425 nm. The TFC was calculated based on a standard curve of quercetin (5–35 mg/mL) expressed as mg quercetin equivalent (mg QE/100 g) [38]. The analysis was also performed on the crude extract before drying in the same way that was performed on the microparticle.

#### 2.6.3. Tannin Condensate Content (TCC)

The TTC quantification in the MP 5% was performed by the UV/Vis spectrophotometric method (Shimadzu UV 1800, Kyoto, Japan) by the reaction of 1% vanillin and 8% hydrochloric acid (HCl). Briefly, 5 mL the 1:1 vanillin-HCl mixture was added to 25 mL test tubes (in triplicate) and prewarmed in a water bath at 30 °C for 30 min. Then, 1 mL of the solution obtained from the extraction of the polyphenols content from the microparticle was added and mixed using a vortex for 30 seg. The reaction was kept at 30 °C in a water bath for 30 min followed by measurement of absorbance at 500 nm. The TCC was calculated based on the catechin standard (20–100 mg/mL) expressed as mg catechin equivalent (mg CA/100 g) [39]. The analysis was also performed on the crude extract before drying in the same way that was performed on the microparticle.

### 2.7. Ultraefficiency Liquid Chromatography (UHPLC-DAD) Analysis

Phenolic compounds were detected by UHPLC-DAD in MP 5%. The analyses were performed using a high-efficiency liquid chromatograph (Agilent 1260 infinity, Singapore). The standards used were: gallic acid, caffeic acid, 2-hydroxycinnamic acid, quercetin, epicatechin and rutin. Standard solutions were prepared at a concentration of 0.5 mg/mL for caffeic acid and 2-hydroxycinnamic acid and at 1 mg/mL for gallic acid, epicatechin, quercetin and rutin. The microparticle was prepared at a concentration of 30 mg/mL. The sample and standard were filtered cellulose membranes (0.22 μm particle size) (Milipore, Bedford, MA, USA). An injection volume of 10 μL was used. Separation was achieved on XDB C18 column (4.6 mm × 250 mm 5 μm particle size), maintained at 30 °C. The mobile phase consisted of acidified water 0.1% formic acid (Solvent A) and methanol (Solvent B) at a flow rate of 0.3 mL/min. Gradient elution was executed as follows: the initial ratio was 5% B changed to 15% in 5 min, 40% in 17 min, 60% in 23 min and 90% in 26 min, before returning to the initial ratio in 36 min. The system was equilibrated for 2 min prior to the subsequent analysis. Chromatographic peaks were detected wavelengths in 254 nm, 280 nm, 325 nm and 365 nm [40]. Different polyphenols were identified by comparing the UV absorption spectra, the retention time of samples compared with those of standards and co-injection of the sample with the standards. Cochromatography was performed to confirm the identity of the compounds. Cochromatography was performed with 5 µL aliquots of the caffeic acid and 2-hydroxycinnamic acid solution, 10 µL of the gallic acid and quercetin solution and 20 µL of the epicatechin and rutin solution solvent (methanol). After this period, 1 mL of the solution obtained from the extraction of polyphenols content from the MP 5% was added and injected into the chromatograph under the same conditions mentioned above. The analysis was also performed on the crude extract (30 mg) before drying in the same way that was performed on the microparticles.

### 2.8. 2,20-Azinobis (3-Ethylbenzothiazoline-6-Sulfonic Acid) (ABTS) Cation Scavenging Activity

Antioxidant activity by the free radical scavenging ABTS was performed in a spectrophotometer (Shimadzu^®^ UV 1800, Kyoto, Japan). The ABTS radical was created by adding 5 mL ABTS (7 mM) solution to 88 µL of the potassium persulfate solution (1.40 mM). The mixture was kept dark at room temperature for 16 h. Briefly, 30 μL of the solution obtained from the extraction of the polyphenols content from the MP 5% was mixed with 3000 μL of ABTS radical and incubated in the dark for 6 min followed by measurement of absorbance at 734 nm. The antioxidant activity was calculated based on a Trolox standard curve (100–2000 μM) and the results are expressed as μM Trolox equivalent (μM TE/100 g) [41]. The analysis was also performed on the crude extract before drying in the same way that was performed on the microparticles.

### 2.9. 2,2-Diphenyl-1-Picrylhydrazyl (DPPH) Free Radical Scavenging Activity

Antioxidant activity by the free radical scavenging DPPH was performed in a spectrophotometer (Shimadzu^®^ UV 1800, Kyoto, Japan). A solution of 0.06 mM DPPH/methanol was prepared by dissolving 2.4 mg DPPH (2,2-diphenyl-1-picrylhydrazyl) in 100 mL methanol. Briefly, 100 µL of the solution obtained from the extraction of the polyphenols content from the MP 5% was mixed with 3900 µL of DPPH radical and incubated in the dark for 30 min followed by measurement of absorbance at 515 nm. The antioxidant activity was calculated based on a DPPH standard curve (10 µM–60 µM), and the percent inhibition was calculated according to Equation (2) [42]. The IC_50_ value was defined as the final concentration expressed as μg/mL of the extract capable of reducing the initial concentration of 50% DPPH. The analysis was also performed on the crude extract before drying in the same way that was performed on the microparticle.

(2) $$\%\;\mathrm{inhibition}=\frac{\;\mathrm{Abs}\;\mathrm{DPPH}\;–\;\mathrm{Abs}\;\mathrm{sample}}{\mathrm{Abs}\;\mathrm{DPPH}}\;\times 100$$

### 2.10. Physicochemical Analysis

#### 2.10.1. Infrared Analysis (FTIR)

The FTIR spectrum was obtained by spectrophotometer (IR Prestige-21 Shimadzu^®^ Kyoto, Japan), it was performed by mixing the powder sample with potassium bromide (KBr) and pressing it at high pressure, forming a tablet. The absorption spectrum was analyzed in the range of 4000 to 600 cm^−1^, with 32 scans and a resolution of 4 cm^−1^ [43]. The samples submitted to FTIR analysis were the crude extract, maltodextrin and MP 5%. The spectrum was obtained using the Origin Pro2019 software.

#### 2.10.2. Thermogravimetry Analysis (TG)

The TG curves of the crude extract, maltodextrin and MP 5% were obtained in a TGA-50 thermal analyzer (Shimadzu^®^, Kyoto, Japan) using a platinum crucible with approximately 9.0 mg of sample under a nitrogen atmosphere (N_2_) and flow of 50 mL/min. The experiment was carried out from room temperature to 600 °C and a heating rate of 10 °C/min. The data obtained were analyzed using the TA-50 W Shimadzu^®^ software [44].

#### 2.10.3. Moisture Content

Determination of the moisture content was evaluated by the gravimetric method on a scale for moisture analysis with a halogen lamp (GEHAKA, São Paulo, Brazil). Approximately 2 g of the MP 5% powder was placed on a scale for moisture analysis with a halogen lamp at a temperature of 105 °C for a time of 15 min (in triplicate). The balance determined the exact value of the percentage of moisture loss [34].

#### 2.10.4. Water Activity

Water activity in MP 5% was determined using electronic equipment (Aqua Lab Dew Point 4 TEV, São Paulo, Brazil) at a temperature of 25 °C. The operation was performed in triplicate [45].

#### 2.10.5. Microstructure of Particles

Morphological analysis (evaluated in triplicate) in MP 5% was performed using the Scanning Electron Microscope (Tescan, VEGA 3, Jena, Germany). The microparticle was deposited on a sample holder with the aid of carbon adhesive tape and coated with a 15 nm thick layer of gold (Au) for 1.5 min and observed in secondary electrons and magnification of 1000× and 5000× [46].

### 2.11. Statistical Analysis

Statistical analysis was conducted by using one-way ANOVA in the BioEstat 5.0 program (Tefé, AM, Brazil). All samples were analyzed in triplicate. Tukey’s test was used to evaluate the significant differences (*p* < 0.01) among the test sample. The results are expressed as mean values and standard deviations.

## 3. Results and Discussion

### 3.1. Quantification of Polyphenols and Encapsulation Efficiency

This work quantified the total polyphenols, flavonoids and condensed tannins content extracted from the coproduct of tucuma almonds. Phenolic content in foods is very variable, which directly affects their individual dietary intake. The biological effects on health induced by phenolic compound intake, either as natural dietary components or as a supplementation using nutraceuticals or functional foods, depend on a great number of factors, such as the cultivar, the climate, the transport conditions, the phenolic compound interactions, the interindividual variability and the circadian rhythm [47].

The values of total polyphenols, total flavonoids and condensed tannins were higher than expected (Table 1). Considering that in this work we used the waste of the tucuma, we can infer that the polyphenols content found in our samples is relevant. Another fact to be considered, highlighting the relevance of the data found in this study, is that the content of phenolic compounds (total polyphenols and total flavonoid) is concentrated in the peel and pulp of the fruit. 

Sagrillo et al. [48] performed the extraction of the content of phenolic compounds from the peel and pulp of tucuma (*Astrocaryum aculeatum* Meyer) and obtained for the pulp values the total polyphenols (426.35 mg/100 g), total flavonoids (26.06 mg/100 g) and condensed tannins (4.03 mg/100 g) as well as the peel’s total polyphenols (790.95 mg/100 g), total flavonoids (77.52 mg/100 g) and condensed tannins (26.37 mg/100 g). The results found in this study (Table 1) demonstrate that the coproduct of tucuma almonds are sources of phenolic compounds, corroborating the study by Gonçalves et al. [49], who evaluated the amount of polyphenols in 22 Brazilian fruits, 10 of which are native to the Amazon, including tucuma, and the results point out that tucuma is one of the main sources of polyphenols among the most consumed fruits in the Amazonian diet. Differences in the total polyphenols, total flavonoids and condensed tannins content of the samples are statistically significantly different (*p* < 0.01); the crude extract of the coproduct showed a total of 4.4 mg, 4.6 mg and 11.6 mg more than the total polyphenols, total flavonoids and condensed tannins, respectively, contained in the microparticle (Table 1). The difference may be associated to a small loss by phenolic compounds degradation during the drying process [14]. Condensed tannins showed greater losses compared with total polyphenols and total flavonoids, suggesting that they are more susceptible to degradation.

Spray drying has been highlighted when it comes to procedures involving the microencapsulation of bioactive compounds because removing water from products ensures microbiological stability, pH, prevents degradation and oxidation reactions, protects from moisture and reduces the cost of storage and transport, in addition to obtaining a product with specific properties, such as instant solubility [50,51]. After performing the spray dryer microencapsulation process, parameters such as microencapsulation efficiency are important to be evaluated to verify the efficiency of the process. The encapsulation efficiency was calculated as shown in Table 2. According to the encapsulation efficiency analysis, the value shows that the phenolic compounds remained in more than 80% of the microparticles after the drying process (Table 2). This means that of the total of 130.5 mg/g total phenolic content, 126.06 mg/g is inside the microparticle or adsorbed in its wall. O the total of 27.17 mg/g of flavonoid content, 22.55 mg/g is inside the microparticle or adsorbed in its wall. Of the total of 62.07 mg/g of tannin condensate content, 50.50 mg/g is inside the microparticle or adsorbed in its wall.

Paini et al. [14], in their study that evaluated the parameters for microencapsulation of phenolic compounds from olive pomace, obtained the highest microencapsulation yield with 10% maltodextrin. Gabbay Alves et al. [19] optimized a study for the microencapsulation of phenolic compounds of the cocoa coproduct almonds using the response surface methodology and obtained optimal conditions with MD 5.0% maltodextrin. Da Costa et al. [18] performed an optimization study for the microencapsulation of phenolic compounds of the cupuassu coproduct almonds using the response surface methodology and obtained optimal conditions with 5.0% maltodextrin. Blagojevi’c, B. et al. [52] encapsulated with 20% maltodextrin the blackthorn (*Prunus spinosa* L.) extract rich in phenolic acids and anthocyanins and demonstrated an encapsulation efficiency of 99.93%.

There is an optimum concentration of the material to be encapsulated as a way to guarantee greater yield [53]. The type of wall material and its quantityare some of the main factors influencing the microencapsulation process of bioactive compounds in relation to the protection, stability and application of the product [54]. The results obtained in this study suggest that 5% of the encapsulating agent was sufficient to protect the phenolic compounds from large degradation losses after the drying process, which was confirmed in the encapsulation efficiency.

The high content of phenolic compounds in industry coproducts, such as seeds and peels, makes these matrices interesting from the point of view of their possible use as flour. The incorporation of coproducts into bakery products can generate health benefits. Foods such as breads and pasta can be enriched with the partial replacement of flour by the flour of the coproducts, as it is an ingredient rich in fiber, proteins, carbohydrates and lipids and represents a rich source of bioactive compounds to obtain healthy formulations [55].

### 3.2. Ultraefficiency Liquid Chromatography (UHPLC-DAD) Analysis

Polyphenols detection was carried out using UHPLC-DAD (six standards were used). The identification and attribution of the peaks of the phenolic compounds present in the crude extract and microparticle were based on the comparison of their retention times and DAD spectra with those of the reference standards and confirmed with the co-injection. The chromatograms obtained with the standards show a peak at retention time of 8.51 min (280 nm) for gallic acid and a peak at the time of 18.51 min (325 nm) for caffeic acid. The retention time of the gallic and caffeic acid peaks detected in the crude extract and microparticle were very similar to the retention times of the standards (Table 3).

**Figure 1:**
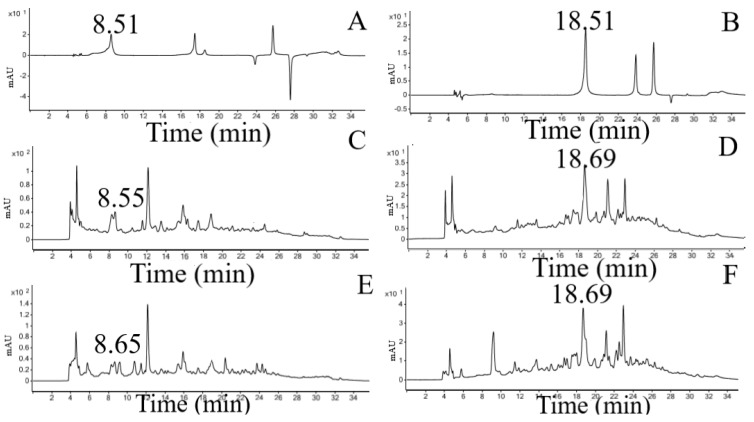
Chromatograms obtained for standards, crude extract and microparticle. (**A**) Gallic acid standard (280 nm) and (**B**) caffeic acid standard (325 nm). (**C**) Peak gallic acid in crude extract (280 nm). (**D**) Peak caffeic acid in crude extract (325 nm). (**E**) Peak gallic acid in microparticle (280 nm). (**F**) Peak caffeic acid in microparticle (325 nm).

Chromatograms obtained from the standards, crude extract and microparticle (280 nm and 325 nm) are shown in Figure 1. In the chromatograms obtained for crude extract and microparticle at 280 nm, a peak with the retention time of 8.55 min and 8.65 min for gallic acid is observed, similar to that observed for the standard for gallic acid (Rt 8.51 min). In the chromatograms obtained at 325 nm, a peak in the retention time of 18.69 min was observed in the crude extract and microparticle, similar to the peak of the standard caffeic acid (Rt 18.51).

**Figure 2:**
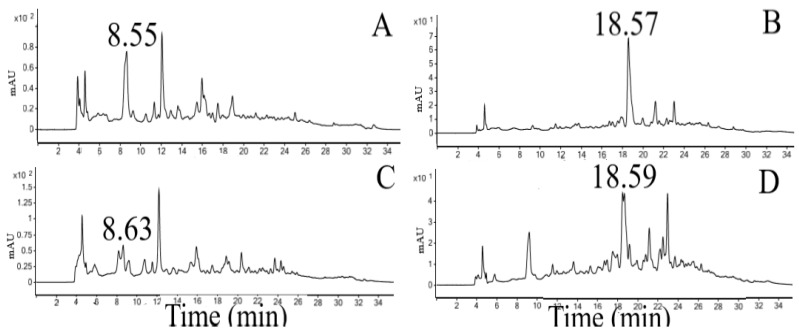
Chromatograms obtained for crude extract and microparticle after co-injection with the standard. (**A**) Peak gallic acid in crude extract (280 nm). (**B**) Peak caffeic acid in crude extract (325 nm). (**C**) Peak gallic acid in microparticle (280 nm). (**D**) Peak caffeic acid in microparticle (325 nm).

The addition (co-injection) of the standard substance in the crude extract and microparticle promoted an increase in the peak area, which suggests that it is gallic acid, recorded in 8.55 min in crude extract and 8.63 in microparticle, and caffeic acid, recorded in 18.57 min (crude extract) and 18.59 min (microparticle) (Figure 2).

**Figure 3:**
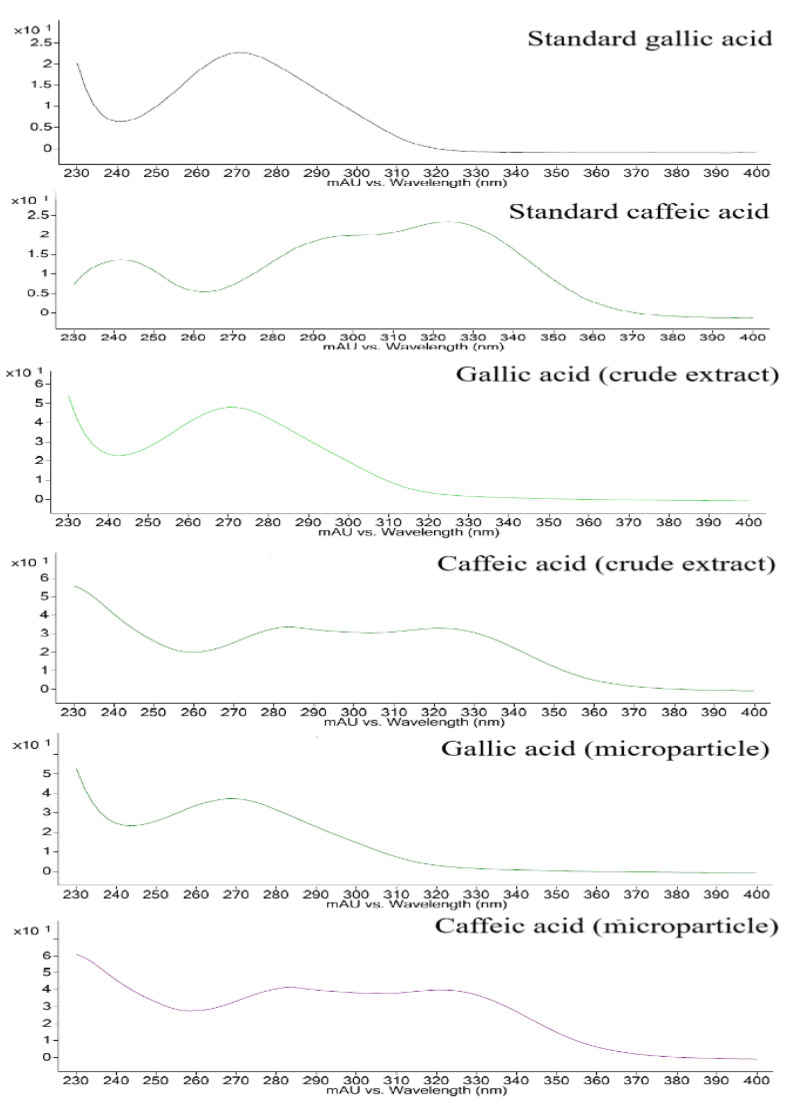
Spectrum of the standard of gallic acid, caffeic acid and spectrum referring to these substances in the crude extract and microparticle.

Figure 3 shows the spectrum obtained from the standard of gallic acid and caffeic acid and the spectrum related to these substances in the crude extract and microparticle.

**Figure 4:**
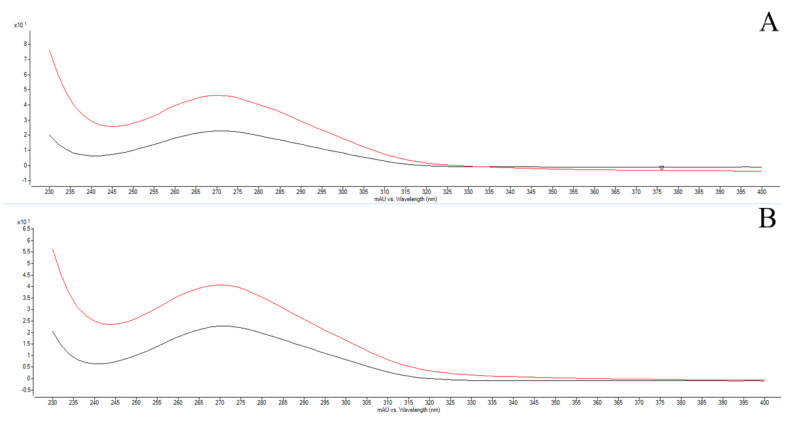

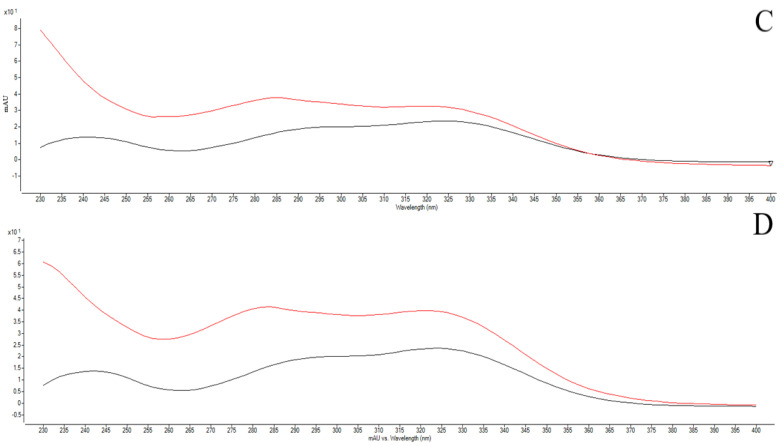
Superposition of the spectra of the patterns and substances found in the crude extract and microparticle. (**A**) In red spectrum is the standard of gallic acid and black spectrum refers to the gallic acid in crude extract. (**B**) In red spectrum is the standard of gallic acid and black spectrum refers to the gallic acid in microparticle. (**C**) In red spectrum is the standard of caffeic acid and black spectrum refers to the caffeic acid in crude extract. (**D**) In red spectrum is the standard of caffeic acid and in black spectrum refers to the caffeic acid in the microparticle.

After the similarity of the retention peaks of the gallic acid and caffeic acid standard and the peaks referring to these substances in the crude extract and in the microparticle, the spectrum was superimposed at these times (Figure 4).

Next, comparing the chromatograms of samples with that of standards, the gallic and caffeic acid were detected. The chromatograms also showed several other peaks that may be related to the presence of flavonoids, glycosylated compounds and other phenolic acids. It is worth mentioning that the sample is an extract (complex mixture), which makes it difficult to obtain a good separation of the peaks and obtain characteristic spectra [56]. In this perspective, phenolic acids have gained momentum owing to their immense dietary health benefits and functionalities such as antioxidant, anti-inflammatory, immunoregulatory, antiallergenic, antiatherogenic, antimicrobial, antithrombotic, cardioprotective and anticancer activities and antidiabetic properties [57]. The presence of gallic and caffeic acid was reported in another species of the genus Astrocaryum that identified and quantified the phenolic compounds by HPLC in the peel and pulp of the tucuma (*Astrocaryum aculeatum*). At the time, chlorogenic acid, flavonoids (rutin and quercetin) and β-carotene [48] were also identified and quantified. The identification of the gallic acid and caffeic acid in the microparticle confirms that maltodextrin protected these compounds during the drying process and corroborates the result of the quantification of phenolic compounds by spectrophotometry.

### 3.3. Antioxidant Activity

Much research has revealed that phenolic compounds are mainly responsible for the antioxidant activity in fruits [53,58,59,60,61,62]. The antioxidant activity was performed by the free radical scavenging ABTS and free radical scavenging DPPH, and the results are shown in Table 4. The data obtained for both methods showed a statistically significant difference (*p* < 0.01) between samples. Three different concentrations of the crude extract and solution obtained from the extraction of polyphenols content from the microparticle were evaluated for both methods (10 μg/mL, 25 μg/mL and 40 μg/mL). The crude extract and the microparticle showed good antioxidant activity for the two methods tested. The higher the concentration of the extract, the higher the percentage of inhibition and the lower the IC 50, and consequently, the greater antioxidant activity [63,64].

The microparticle presented a higher percentage of inhibition than the crude extract in both concentrations evaluated by the DPPH method. At the concentration of 10 μg/mL, the microparticle showed an inhibition of 54.28%, four times greater than the crude extract (9.5%), i.e., a difference of more than 44.78% between the microparticle and the extract. At the concentration of 25 μg/mL, the difference in inhibition was smaller, around 21.73%; the microparticle showed inhibition of 61.87%, while the extract 40.14%. Regarding the highest concentration of 40 μg/mL, the difference between the microparticle and extract was only 1.2%. The microparticle showed an inhibition of 83.19% and crude extract 81.99%. In relation to the IC_50_ values, the microparticle’s were lower in both concentrations compared with the crude extract, confirming that the microparticle presented higher antioxidant activity than the extract. The microparticle at the concentration of 40 μg/mL presented an IC_50_ of 24.12 µg/mL, twice that of the extract (60.22 µg/mL), a difference of 36.1 µg/mL. At the concentration of 25 μg/mL the microparticle presented an IC_50_ of 19.37 µg/mL, while the extract was 38.29 µg/mL, with a difference of 18.9 µg/mL between them. At the concentration of 40 μg/mL, the microparticle presented a value of IC_50_ of 5.91 µg/mL and the extract 3.29 µg/mL, a difference of 2.38 µg/mL. The increase in antioxidant activity in the microparticle in relation to the extract may be related to the greater number of hydrogen donor hydroxyls present in the microparticle.

Crude extract at a concentration of 10 μg/mL showed an inhibition percentage of less than 10% and did not show a reduction capacity of 50% of the DPPH radical. Only from a concentration of 25 μg/mL was it able to reduce the radical by 50%. The concentration of 40 μg/mL showed an inhibition percentage of more than 80%. The microparticle evidenced that the concentration of 10 μg/mL is capable of reducing the DPPH radical by 50%. It also showed higher percentages of inhibition than the crude extract in the three concentrations. The IC_50_ values were lower in the microparticle in relation to the crude extract. The greater the amount of hydrogen donor hydroxyl present in the sample, the greater its antioxidant activity. The antioxidants present in the extract and in the microparticle were able to donate hydrogen radicals to DPPH [65].

In the evaluation of the antioxidant activity by the ABTS radical method, the highest concentration of the crude extract and solution obtained from the extraction of polyphenols content from the microparticle (40 μg/mL) showed the highest antioxidant activity, which was also observed for the DPPH radical. However, the crude extract presented superior results in relation to the microparticle, different from what was verified in the evaluation by DPPH. The antioxidants present in the crude extract and in the microparticle captured the ABTS cation, promoted radical inhibition and produced antioxidant activity. At the concentration of 10 μg/mL, the crude extract presented a value of 1094.01 µM trolox and the microparticle 938.91 µM trolox, a difference of 155.10 µM trolox. At the concentration of 25 μg/mL, the extract showed a value of 1101.33 µM trolox and the microparticle 941.33 µM trolox, with a difference of 160 µM trolox between them. In relation to the concentration of 40 μg/mL, the value obtained for the extract was 1247.88 µM trolox and the microparticle 956.01 µM trolox, with a difference of 291.87 µM trolox between them. The higher antioxidant activity of the crude extract in relation to the microparticle is justified because there was a decrease in phenolic compounds after the drying process and consequently the antioxidant activity in the microparticle was also reduced. Ferreira, L et al. [3] performed the microencapsulation by spray drying the oily extract of the coproduct the tucuma almond and found good antioxidant activity for the ABTS (537.12 ± 0.01 µM trolox) and β-Carotene/Linoleic Acid (43.3 ± 2.3%) methods.

The good antioxidant activity found in the tested methods confirms that the concentration of 5% maltodextrin protected phenolic compounds from high degradation losses and maintained the activity. Additionally, good antioxidant activity found in the coproduct of tucuma is necessary and is of great importance, as it may be an indication of its possible use to improve the nutritional and functional values of food products. The presence of antioxidant compounds, if added to some perishable foods, together with the increase in nutritional value, can delay the oxidation process of lipids, thus prolonging the shelf life of products. In addition, in accordance with national and international guidelines related to waste management, the main strategies for an effective management and sustainability system for the food industry emphasize the importance of waste prevention/minimization, as well as the recovery and promotion of coproducts [55].

### 3.4. Infrared Analysis (FTIR)

**Figure 5:**
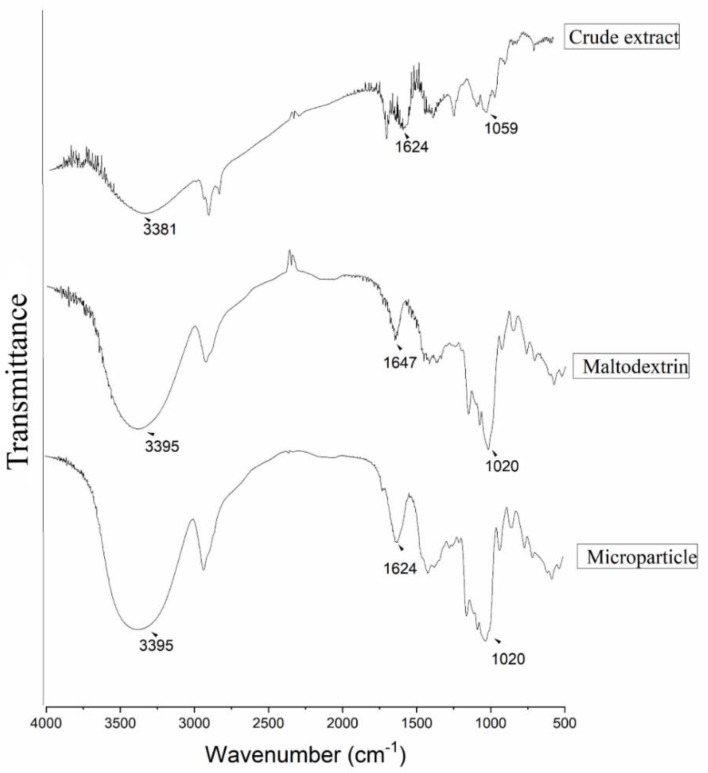
FTIR spectra of the crude extract, maltodextrin adjuvant and microparticle in the absorption range of 4000 to 600 cm^−1^, with 32 scans and 4 cm^−1^ resolution.

Toward the investigation of the extract–maltodextrin chemical interactions and characterization of the microparticle, the samples were tested by FTIR. The FTIR spectra of the crude extract, maltodextrin and microparticle are shown in Figure 5. The spectra obtained from the crude extract shows bands in 3381 cm^−1^ characteristic O–H stretch vibration of alcohols and phenols. The peak 1624 cm^−1^ may be related to C=O stretching the vibration of carbonyl present in [3,66]. The absorption range at 1059 cm^−1^ is related to vibrations of the C–O stretch of phenols and alcohols. However, the spectrum obtained for the microparticle and maltodextrin showed bands in 3395 cm^−1^ O–H stretch vibration, characteristic of alcohols and phenols. Absorption bands in 1647 cm^−1^ can be attributed to the C=O stretching vibrations of acid carbonyl. Absorption peaks in 1020 cm^−1^ are relative to C–O stretching vibrations of alcohol (Figure 5) [35].

The analysis of characteristic bands of certain functional groups of a molecule provides, through a simple examination of the spectrum and consultation of tables and data, a set of information on the structure of the molecule [67]. The spectra obtained in this work presented an aggregate of absorption bands related to stretches C=O, C–O and O–H that may be related to important functional groups such as alcohols, phenols, alkanes, alkenes, methyl, amides present in the structure of phenolic compounds and anhydroglucose ring, corresponding to maltodextrin [23]. In the fingerprint region, it is possible to see great similarity between the bands in maltodextrin and microparticle spectrum; this may be due to the encapsulation of the extract in the microparticle. The spectrum of the microparticle is similar to the spectrum of maltodextrin, this may be due to the overlap of the extract due to the formation of the microsphere. The atomization of the extract using maltodextrin as a wall material generated microspheres; this design justifies the nonalteration of the spectrum obtained for the microparticle [44]. Therefore, FTIR contributes as a structural characterization technique in order to confirm the presence of important functional groups’ crude extract and in the microparticle.

### 3.5. Thermogravimetry Analysis (TG)/Derived Thermogravimetry (DTG)

**Figure 6:**
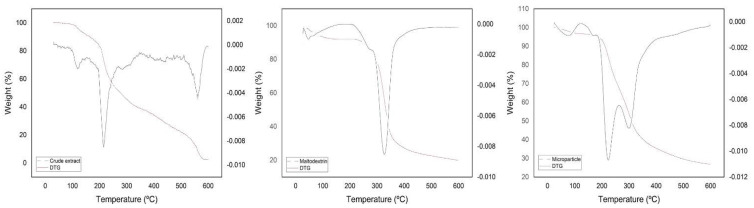
Thermogravimetry analysis (TG) (−)/derived thermogravimetry (DTG) (−) curve of crude extract, microparticle and maltodextrin adjuvant. Conditions: nitrogen atmosphere (N_2_), the flow of 50 mL/min and a heating rate of 10 °C/min.

Figure 6 shows the TG / DTG curves of the crude extract, maltodextrin and microparticle. The crude extract showed three mass-loss events. The first occurred in the range of 109.52–206 °C with a mass loss of 17.30%. The second event occurred in the range of 206.94–495.64 °C, with a mass loss of 58.16%. The third occurred in the interval between 496.24–588 °C with a mass loss of 20.14%. Plant extracts are complex mixtures of organic substances with different boiling points and different thermal stability due to chemical and physicochemical differences among these substances. Thermogravimetry has no specificity, so it can only be inferred that the first event of loss of mass is due to the degradation or boiling of simple molecules of low molecular weight, the second and third events occur due to the “exit” of more complex substances, such as phenolic compounds, and the degradation of high molecular weight proteins, among other substances that may be present in the extract [3,22,26,68]. Altogether, the crude extract lost 95.60% of its initial mass, leaving at the end of the analysis the value of 4.39% of mass that corresponds to the inorganic portion that made up the crude extract.

The microparticle showed three mass-loss events. The first event occurred in the range 49–100 °C with a mass loss of 3.10%. The second event occurred in the range of 200–239 °C with a mass loss of 29.40%. The third event occurred in the range of 283–356 °C with a mass loss of 40.50%. The maltodextrin adjuvant showed only one event occurring in the range 297–350 °C with a mass loss of 66.50%. In an encapsulation study of the blackthorn (*Prunus spinosa* L.) extract, the maltodextrin showed degradation with a mass loss in interval from 300 °C to 350 °C [52]. Maltodextrin is a hygroscopic material, probably, this first event in the microparticle is a consequence of the storage conditions. The storage conditions may have contributed to the increase in moisture in the formulations, and therefore they present a loss of mass in this temperature range of up to 100 °C; the same does not occur in the crude extract TG curves [27,66]. Through the TG curve, the existence of the third event is almost imperceptible, and although the DTG shows the third event, it is observed that between the second and third events the DTG curve cannot return to the base line, and there is no defined limit between these events. This is probably due to the degradation of the maltodextrin and extract at very close temperatures.

The DTG curve shows a second “splited” event; the first “peak”, the second event of the curve, happened in a temperature range similar to that observed in crude extract. Maltodextrin has a melting temperature of 240 °C [66], so it can be considered that at the beginning of the second event the microspheres are in a process of degradation, at least losing their spherical shape due to the melting of the maltodextrin, which could result in a physical mixture between the extract and maltodextrin inside the sample holder. Thus, both suffer the action of temperature independently, and for this reason, the second and third events are seen to occur at temperatures similar to the mass loss events of maltodextrin and crude extract. Through the thermogravimetric study, it was possible to obtain information on the relationship between humidity and maximum temperature stability of the extract and microparticle [69]. Moreover, it was observed in the encapsulation study of the blackthorn (*Prunus spinosa* L.) extract that the mass-loss temperature shift of the encapsulates and crude extract proved that the extract bioactives were protected within the microparticle [52].

### 3.6. Moisture Content and Water Activity

The moisture content and water activity affects the processing capacity, handling properties and stability of the powder [70]. Moisture content values of less than 6% suggest higher stability and the shelf life of food products [71,72]. Reduced values of moisture content and water activity indicate higher stability of the product during storage, and foods that have a moisture content of more than 20% and a higher water activity of 0.60 are subject to deterioration processes caused by molds and yeasts [73]. The values found for the moisture content of 3.57 ± 0.83% and water activity 0.25 ± 0.01 were low and indicate a good drying process [74]. The data also indicate that the operating conditions used in drying were sufficient for the evaporation of water [66,75]. The low humidity prevented the formation of the agglomeration phenomena, which decreased the active retention, dispersion and/or flowability of the powder [76,77]. The maltodextrin is a hygroscopic material, the lower the particles’ moisture content, the higher their hygroscopicity, i.e., the greater their capacity to adsorb ambient moisture, which is related toa greater water concentration gradient between the product and the surrounding air [50,51,78].

Formulations with higher concentration of maltodextrin generate a lower percentage of hygroscopicity [79,80,81,82]. The microparticle was prepared with only 5% maltodextrin for the reason of low humidity value. This result suggests that the microparticle may have a longer storage life because it does not have water available for microbial growth and for the occurrence of enzymatic reactions [70]. These values were similar to the study of the microencapsulation of oily extract coproduct of the tucuma almond (water activity 0.25 ± 0.007 and moisture content 6.6 ± 0.06%) [3]. These data were lower than the study of the microencapsulation of extract coproduct of cocoa beans (moisture content varied between 10.35 ± 0.133% and 20.77 ± 0.384%) [19] and the microencapsulation of cupuassu seed coproduct extract (moisture content varied between 10.73 ± 0.78% and 28.00 ± 0.95%) [18].

### 3.7. Microstructure of Particles

**Figure 7:**
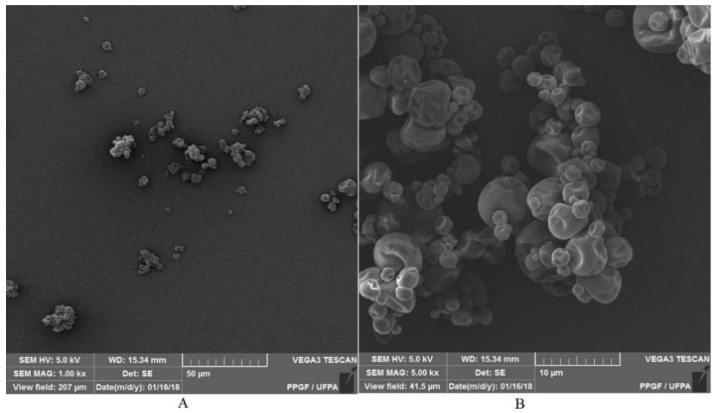
Microparticle photomicrographs: (**A**) (1000× magnification), (**B**) (5000× magnification).

A morphological analysis was carried out to evaluate the characteristics of the microparticles, such as shape and surface (Figure 7). Their external surfaces presented no fissures and thus did not lead to rupture, which is fundamental to guarantee the greater protection of the asset. Microphotographs generally showed spherical particles of heterogeneous sizes and without agglomeration, which supposes that there is a repulsion due to negative charges. It can also be seen that there are microparticles with a smooth surface and others with a wrinkled surface.

The wrinkling can be attributed to the drying and cooling process of the particles in the spray dryer [80]. This roughness is probably influenced by the drying speed and the viscoelastic properties of the wall material [54]. The particles showed heterogeneity and were polydispersed, and therefore suggest a greater variation in their properties, mainly in the solubility of the particles in a food matrix [83]. The characteristics found in this work were similar to the study of the oily extract coproduct of the tucuma almond [3]. Encapsulation of the blackthorn (*Prunus spinosa* L.) extract with maltodextrin resulted in particles with a smooth surface but of different sizes and shapes, characteristic for this carrier [52].

## 4. Conclusions

The crude extract of the tucuma coproduct was obtained by the percolation method in a 70% hydroethanolic solution as an alternative to the reuse of the coproduct. The crude extract and the microparticle showed significant levels of phenolic compounds and good antioxidant activity by the tested methods. The microparticle showed spherical and heterogeneous structures and good encapsulation efficiency from the spray drying process using maltodextrin as a wall material. The microparticle also had low humidity and water activity, an indication of good stability and conservation. Thus, it is suggested that the extraction and drying processes were efficient and kept the antioxidant activity preserved, generating a product rich in phenolic compounds with possible application in the food area of functional food.

## Figures and Tables

**Table 1:** Quantification of polyphenols content in the crude extract (CE) and microparticle (MP).

Sample	Total Phenolic Content (mg GAE/100 g)	Total Flavonoid Content (mg QE/100 g)	Tannin Condensate Content (mg CA/100 g)
Crude extract	135.1 ± 0.078 ^a^	32.73 ± 0.009 ^a^	76.29 ± 0.001 ^a^
Microparticle	130.5 ± 0.024 ^b^	27.17 ± 0.002 ^b^	62.07 ± 0.002 ^b^

Experiments were executed in triplicate and the data are presented as mean ± standard deviation (SD). Different letters in the same column indicate significant difference (*p* < 0.01). GAE = gallic acid equivalent, QE = quercetin equivalent and CAE = catechin equivalent.

**Table 2:** Values obtained from the encapsulation efficiency of phenolic compounds in the microparticle (MP 5%).

Encapsulation Efficiency (%)	
Total Phenolic Content	96.60 ± 0.10
Total Flavonoid Content	83.01 ± 0.01
Tannin Condensate Content	81.36 ± 0.01

Experiments were executed in triplicate and the data are presented as mean ± standard deviation (SD).

**Table 3:** Polyphenols identified in the crude extract and microparticle with the identification of the retention time of each peak and their respective wavelengths.

	CE RT (min) Area		MP 5% RT (min) Area		Standards RT (min) Area	
Gallic acid (280 nm)	8.55	909.86	8.65	423.70	8.51	647.74
Caffeic acid (325 nm)	18.69	537.95	18.69	822.01	18.51	462.07

CE = crude extract; MP = microparticle; RT = retention time.

**Table 4:** Antioxidant activity in the crude extract and microparticle by DPPH and ABTS.

Sample	DPPH			ABTS^+^ (µM Trolox)
	Conc Inhibition (%)		IC_50_ (µg/mL)	
	10	9.5 ± 0.52 ^a^	60.22 ± 0.86 ^a^	1094.01 ± 7.33 ^a^
Crude extract	25	40.14 ± 0.31 ^a^	38.29 ± 0.24 ^a^	1101.33 ± 3.66 ^a^
	40	81.99 ± 0.50 ^a^	8.29 ± 0.80 ^a^	1247.88 ± 3.60 ^a^
Microparticle	10	54.28 ± 0.97 ^b^	24.12 ± 0.61 ^b^	938.91 ± 4.23 ^b^
	25	61.87 ± 1.25 ^b^	19.37 ± 0.79 ^b^	941.35 ± 6.23 ^b^
	40	83.19 ± 1.21 ^b^	5.91 ± 0.76 ^b^	956.01 ± 7.63 ^b^

Values mean ± standard deviation (*n* = 3); different letters in the same column indicate significant difference (*p* < 0.01).

## Data Availability

All data generated or analyzed during this study are included in this article.

## Sample Availability

Samples of the compounds are available from the authors.
